# Madecassoside Inhibits Body Weight Gain *via* Modulating SIRT1-AMPK Signaling Pathway and Activating Genes Related to Thermogenesis

**DOI:** 10.3389/fendo.2021.627950

**Published:** 2021-03-09

**Authors:** Boju Sun, Misa Hayashi, Maya Kudo, Lili Wu, Lingling Qin, Ming Gao, Tonghua Liu

**Affiliations:** ^1^Second Clinical Medical College, Beijing University of Chinese Medicine, Beijing, China; ^2^School of Pharmaceutical Sciences, Mukogawa Women’s University, Hyogo, Japan; ^3^Key Laboratory of Health Cultivation of the Ministry of Education, Beijing University of Chinese Medicine, Beijing, China; ^4^Technology Department, Beijing University of Chinese Medicine, Beijing, China; ^5^Institute for Biosciences, Mukogawa Women’s University, Hyogo, Japan

**Keywords:** madecassoside, obesity, UCP-1, SIRT1-AMPK signaling pathway, brown fat

## Abstract

**Background:**

Pre-clinical research studies have shown that Madecassoside (MA) has favorable therapeutic effects on arthritis, acne, vitiligo and other diseases. However, the effects of MA on obesity have not yet been studied. This study mainly aimed to investigate the effects of MA in protecting against obesity and its underlying mechanism in reducing obesity.

**Methods:**

Obese diabetic KKay/TaJcl mice model was adopted to the study. The body weight of all animals was recorded daily, and the blood glucose, blood lipid, and serum aminotransferase levels were examined, respectively. The expression of P-AMPK, SIRT1, P-LKB1, P-ACC, and P-HSL in abdominal fat, mesenteric fat, and epididymal fat was measured by western blotting, and the levels of PPARα, CPT1a, PGC-1α, UCP-1, Cidea, Cox7a1, and Cox8b were examined by real-time quantitative PCR (RT-qPCR).

**Results:**

The results revealed that the body weight of the mice in MA group was significantly reduced, and the body mass index (BMI) showed significant difference between the two groups after 8 weeks of MA treatment. Further research revealed that it affected the mesenteric fat and epididymis fat by activating SIRT1/AMPK signaling pathway, and then promoted fatty acid oxidation of epididymal fat (PPARα ↑, CPT1a↑, and PGC-1α↑). Last but not the least, it also promoted the expression of UCP-1 and stimulated thermoregulatory genes (Cidea, Cox7a1, and Cox8b) in brown fat and mesenteric fat.

**Conclusions:**

Taken together, these findings suggest that MA can inhibit the weight gain in obese diabetic mice, and reduce triglyceride levels, inhibit lipogenesis of mesenteric fat, promote epididymal fat lipolysis and fatty acid oxidation. Furthermore, MA treatment might promote mesenteric fat browning and activate mitochondrial function in brown fat as well as mesenteric fat.

## Introduction

Obesity is a significant risk factor for many diseases, and is a major global health concern. Several large epidemiological and clinical or pre-clinical data have shown that obesity is closely related to the occurrence of various cardiovascular (1), insulin resistance (2), metabolic syndrome (3) and other diseases, seriously affecting the physical health condition of the individuals. *Michael D Jensen* has pointed out that visceral fat storage is closely related to fatty acid metabolism, and its selective dysregulation might play a crucial role in metabolic disorders of obesity (4).

Obesity is mainly treated through lifestyle management (5), drug treatment (6), and even surgical treatment (7). The therapeutic drugs that are currently approved for obesity include orlistat, liraglutide, lorcaserin, etc. Although weight loss is achieved with all these drugs, varying degrees of side effects are inevitable (8). Medicinal plants for obesity and its related complications have attained good curative effects, and have the advantage of fewer side effects. Research and development of medicine plant or plant derived bioactive compounds for obesity is currently the research hot spot.

Previous studies have supported that *Centella asiatica* extract can reverse lipid levels in obese diabetic rats, and it also has a positive effect to inhibit the expression of pro-inflammatory cytokines in 3T3-L1 adipocytes (9, 10). Madecassoside (MA) is a pentacyclic triterpene saponin that is obtained from *Centella asiatica* and possesses multiple pharmaceutical activities. Previous studies have confirmed that MA can be widely distributed to several internal organs, and reach a maximum level within 5–15 min after oral administration, however, the bioavailability of oral intake of MA is poor (less than 1%) (11, 12). Although many beneficial effects associated with MA, such as it can promote wound healing (13), ameliorate lung fibrosis in mice (14), inhibit proliferation and invasion of liver cancer cells (15), prevent neurodegenerative diseases (16), stimulate insulin secretion (17), and relieve osteoarthritis (18) have been reported, research on the effects of MA on obesity has not yet been developed, and so this study aimed to evaluate the interplay between obesity and MA and the possible underlying molecular mechanisms.

## Materials and Methods

### Animals and Treatment

A total of 16 male KKay/TaJcl obese diabetic mice (Japan Clea Co., Ltd, Japan) weighing 35~40 g were randomly divided into two groups, wherein the mice in the control group (n = 8) were administered with water, and those in MA group (n = 8) were administered with MA (40 mg/kg/day) *via* gavage. MA was dissolved in diluted water to obtain an aqueous solution with a final concentration of 0.8 mg/ml, and the daily administration volume was 0.025 ml*g^−1^ body weight. All mice were kept in conventional animal laboratory and housed one mouse per cage, with 12/12 h light-dark cycle, and free access to normal chow diet and water. The body weight of all animals was recorded daily and given a single dose of MA daily through oral gavage for 8 weeks. At the end of week 8, the body mass index (BMI) of each mouse was measured by bioelectrical impedance analysis (BIA) (ImpediVET, ImprdiMed Ltd., Brisbane, Australia). The mice were then anesthetized with isoflurane, and were sacrificed by abdominal aortic blood collection, and the mice tissues were immediately removed (epididymal fat, mesenteric fat, brown fat, abdominal fat, brain, heart, etc.). These were then placed in RNA later™ solution after weighing their weight, and maintained at −80°C for the next analysis. All procedures were conducted in accordance to the guidelines established by the Japanese Physiological Society. This study has been approved by the Experimental Animal Ethics Committee of the Mukogawa Women’s University in Japan (No. P-06-2019-01-A).

### Blood Serum and Organ Weight Analysis

At the 4th week, mice were fasted for 8 h and the blood was drawn from the tail vein for the measurement of serum lipid profiles and blood glucose levels. At the 8th week, the blood samples were collected through abdominal aorta, and the organs from the mice were separated to measure their respective weights immediately. The serum cholesterol (CHO), triglycerides (TG), aspartate aminotransferase (AST), alanine aminotransferase (ALT), and blood glucose levels were detected using a special kit according to the manufacturer’s instructions (Wako Pure Chemical Industries, Ltd., Osaka, Japan).

### Oral Glucose Tolerance Test (OGTT)

Blood was collected from the tail vein of mice after 12 h of fasting. Fasting blood glucose (FBG) was measured using glucose oxidase method at 0, 30, 60, 90, and 120 min after glucose gavage (2 g/kg) in the OGTT. The area under curve (AUC) was then calculated using the following method:

AUC=0.5×(BG 0min+BG 30 min)/2+0.5×(BG 30 min+BG 60 min)/2+1×(BG 60 min+BG 120 min)/2

### RNA Extraction and Reverse Transcription Polymerase Chain Reaction

The RNA of mesenteric fat, epididymal fat, and brown fat was extracted by Sepasol(R)-RNA I Super G (Nacalai Tesque, JAPAN) kit, measured at different absorbance values of 260, 280, and 320 nm, and then the concentration was calculated according to the absorbance value. Next, the RNA was transcribed into cDNA when configuring to 0.6 ug/ul. Specific primers were designed based on the sequence obtained from the literature and screened them through primer-BLAST website (**Table 1**), and then synthesized from Thermo Fisher Scientific (Waltham, MA, USA). Finally, THUNDERBIRD SYBR qPCR Mix was used to amplify the target gene (TOYOBO, Tokyo, Japan), and then all the prepared samples were kept into Thermal Cycler Dice (Takara Bio Inc. Japan). The cycle settings were as follows: 95°C, 30 s, 1 cycle; 95°C, 5 s, 60°C, 30 s, 40 cycles; 95°C, 15 s, 60°C, 30 s, 1 cycle, and the results were analyzed by 2^−ΔΔCt^ method.

**Table 1:** Mouse primer sequences used in qPCR.

Gene	Sense primer (5’-3’)	Antisense primer (5’-3’)
PPARα	ACGCGAGTTCCTTAAGAACCTG	GTGTCATCTGGATGGTTGCTCT
CPT-1a	TATGTGAGGATGCTGCTTCC	CTCGGAGAGCTAAGCTTGTC
PGC-1α	TTCAAGATCCTGTTACTACT	ACCTTGAACGTGATCTCACA
UCP-1	GTGAAGGTCAGAATGCAAGC	AGGGCCCCCTTCATGAGGTC
Cidea	TCC TCG GCT GTC TCA ATG	GGC TGC TCT TCT GTA TCG
Cox7α1	AGG ACG CAA AAT GAG GGC	TCT TGT GGG GGA AGG AGG
Cox8b	GGA GTG CGA CCC CGA GAA T	CGG CGG AAG TGG GAG TTT T
SREBP1c	CAAGAAGCGGATGTAGTCG	GAGCCGTGGTGAGAAGC
FAS	AGCTGCCAGAGTCGGAGAAC	TGTAGCCACGAGTGTCTCG
B-actin	CTTTGCAGCTCCTTCGTTGC	ACGATGGAGGGGAATACAGC
GAPDH	AGAACATCATCCCTGCATCCA	CCGTTCAGCTCTGGGATGAC

### Western Blotting Analysis

Total tissue protein was extracted with homogenized buffer [50 mM Tris-HCl (pH 7.4), 100 mM NaCl, 1% Nonidet-P40, 0.25% sodium deoxycholate, 0.1% sodium dodecyl sulfate (SDS), 1 mM B Extracted protein and acid (EDTA), 50 mM NaF, 2 mM Na3VO4, 30 mM sodium pyrophosphate, 2 mM phenylmethanesulfonyl fluoride (PMSF), 1 mM Benzoidine, 0.02 mg/ml trypsin inhibitor, 0.02 mg/ml leupeptin, and 0.02 mg/ml aprotinin]. The protein concentration was determined by BSA protein determination kit (Bio-Rad), and different proteins were separated by sodium dodecyl sulfate polyacrylamide gel electrophoresis (SDS–PAGE), and then were transferred onto the polyvinylidene fluoride (PVDF) membrane (Amersham Life Science, Inc., Buckinghamshire, UK). The samples were then blocked for half an hour with blocking-one when the above steps were finished. Different primary antibodies were used to incubate the samples at 4°C for overnight. The primary antibodies are as follows: Sirt1 (1/1,000, dilution), P-LKB1 (1/500, dilution), LKB1 (1/1,000, dilution), P-AMPK (1/500, dilution), AMPK (1/500, dilution), P-ACC (1/500, dilution), ACC (1/500, dilution), P-HSL (1/1,000, dilution), HSL (1/1,000, dilution), FAS (1/1,000, dilution), β-actin (1/1,000, dilution) (**Table 2**). β-actin was selected as anti-mouse secondary antibody (1/10,000, dilution), and other primary antibodies were selected by combining with anti-rabbit (1/2,000, dilution). After incubating at room temperature for 1 h, the PVDF membranes were washed with TTBS thrice for 10 min each. And the image results were analyzed using Image J software.

**Table 2:** Antibodies.

Antibodies	Source	Item No.
Sirt1	CST	**9475S**
P-LKB1	CST	**3482**
LKB1	CST	**3047**
P-AMPK	CST	**2535**
AMPK	CST	**5831**
P-ACC	CST	**11818**
ACC	CST	**3676**
P-HSL	CST	**4139**
HSL	CST	**4107**
FAS	CST	**3180**
β-actin	CST	**4970**

These bold values means Item numbers of antibodies that purchased on the CST website.

### Statistical Analysis

The data are expressed as mean ± SD (standard deviation). The data were analyzed using t-test or one-way ANOVA. GraphPad Prism V6.0 software was used to draw the chart. P values of less than 0.05 were considered to be statistically significant different between the groups.

## Results

### MA Inhibits Weight Gain and Decreases BMI in KKay/TaJcl Mice, But Has No Effect on Organ Weight

**Figure 1:**
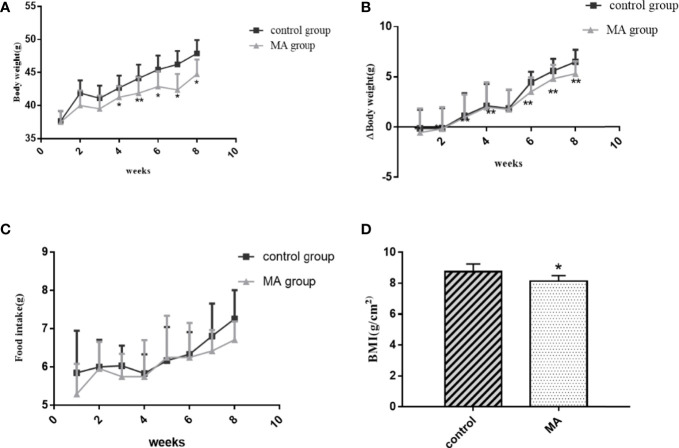
Effect of MA (40 mg/kg/day) on body weight, body weight gain, food intake, and BMI of obese diabetic mice. **(A)** Weekly assessment of body weight in obese diabetic model mice KKay/TaJcl. **(B)** Comparison of body weight gain to that before MA administration. **(C)** Weekly assessment of food intake in obese diabetic model mice KKay/TaJcl. **(D)** BMI values of mice in the two groups. *P < 0.05, **P < 0.01, *vs* control group, and the data are expressed as mean ± SD (n = 8).

The results showed that the body weight of KKay/TaJcl mice was increased every week without any intervention, but the mice in the MA group showed a significant decrease in weight gain. There was a significant difference in weight inhibition when compared to control group at week 4, which continued until the end of the experiment (**Figure 1A**). Also the results of body weight gain as compared to before administration showed a significant difference between the MA group and the control group from week 1 (**Figure 1B**). Food intake results suggested that this dose of MA did not significantly inhibit animals’ appetite compared with that of mice in control group (**Figure 1C**). At the end of the experiment, the BMI of mice in the two groups showed (**Figure 1D**) that MA treatment has effectively reduced the BMI of obese mice. However, the weight of the organs showed no significant difference between the two groups (**Table 3**). These results showed that MA treatment has effectively inhibited weight gain and decreased BMI of KKay/TaJcl mice.

**Table 3:** Effects of MA on the weight of the organs.

	Control (%)	MA (%)
Liver (g)/BW (g) ratio	5.17 ± 0.66	4.87 ± 0.60
Kidney (left) (g)/BW (g) ratio	0.65 ± 0.04	0.65 ± 0.04
Kidney (right) (g)/BW (g) ratio	0.68 ± 0.06	0.7 ± 0.06
Heart (g)/BW (g) ratio	0.4 ± 0.06	0.39 ± 0.06
Epididymal fat (g)/BW (g) ratio	3.13 ± 0.40	3.06 ± 0.41
Brain (g)/BW (g) ratio	0.72 ± 0.07	0.73 ± 0.03

Male obese diabetic model mice KKay/TaJcl were treated with MA or water for 8 weeks. The data are expressed as means ± SD (n = 8). BW, body weight.

### MA Reduces TG Levels, But Has No Effect on CHO and Blood Glucose

**Figure 2:**
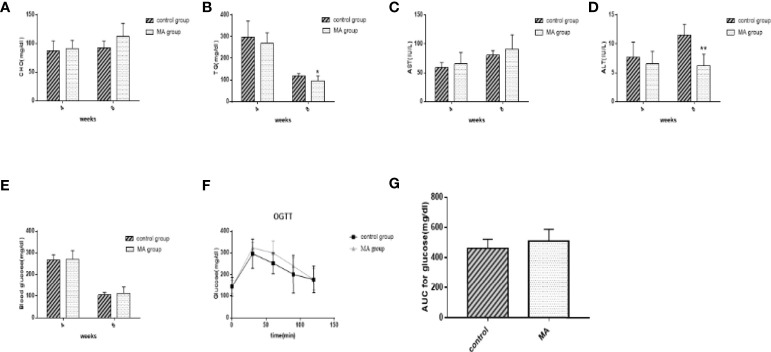
Effects of MA on blood lipid, blood glucose, AST, and ALT levels measured using blood from tail vein at the 4th week and blood from the abdominal aorta at the 8th week. **(A)** Serum total cholesterol levels at weeks 4 and 8. **(B)** Serum TG levels at weeks 4 and 8. **(C, D)** Serum AST and ALT levels at weeks 4 and 8. **(E)** Blood glucose levels at weeks 4 and 8. **(F, G)** Blood glucose levels and AUC in OGTT. *P < 0.05, **P < 0.01, *vs* control group, and data are expressed as mean ± SD (n = 8).

The levels of TG, CHO, AST, ALT, and fasting blood glucose were examined in mice at weeks 4 and 8, respectively, OGTT was conducted before the sacrifice (**Figure 2**). The results showed no significant differences between the two groups during initial MA treatment. Effective reduction of TG was observed, but no significant effect was observed on CHO and fasting blood glucose levels after 8 weeks of intervention. Moreover, the results of AST and ALT indicated that MA does not have any negative effect on the liver function of the mice (**Figures 2C, D**). More importantly, our results showed that MA seemed to not improve Islet function in KKay/TaJcl mice (**Figures 2F, G**). These results indicate that MA might have a certain effect on lowering the lipid levels, whereas no effect on blood glucose levels.

### MA Inhibits Mesenteric Fat Lipogenesis and Promotes Epididymal Fat Lipolysis

**Figure 3:**
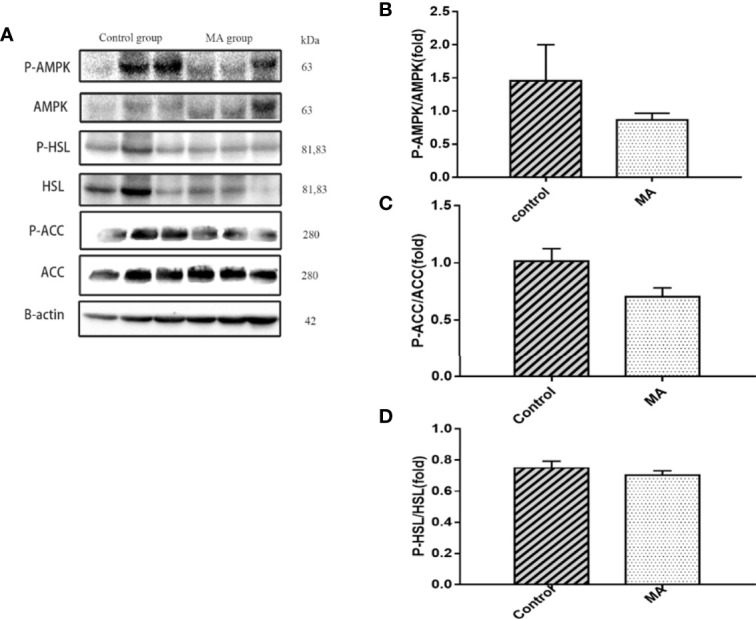
Effects of MA on related factors of abdominal fat. **(A)** The protein expression levels of P-AMPK, AMPK, P-ACC, ACC, P-HSL, HSL, and β-actin of control group and MA group. **(B–D)** The bar graphs indicate the average of P-AMPK, P-ACC, and P-HSL levels. The data are expressed as mean ± SD (n = 8).

**Figure 4:**
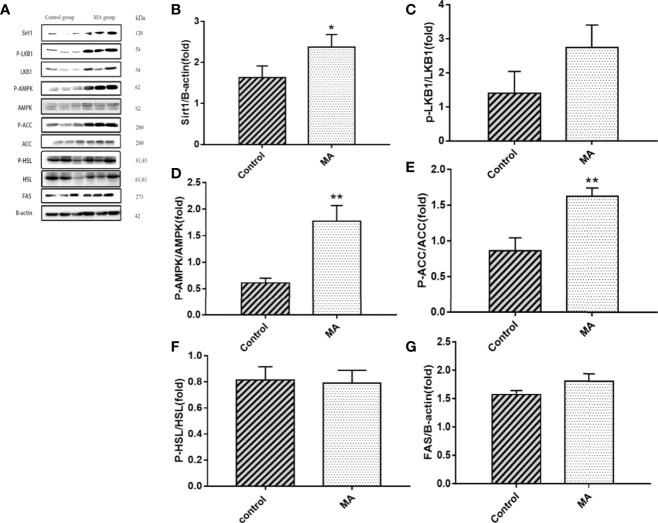
Effects of MA on related factors of mesenteric fat. **(A)** The protein expression levels of SIRT1, P-LKB1, LKB1, P-AMPK, AMPK, P-ACC, ACC, P-HSL, HSL, FAS, and β-actin of control group and MA group. **(B–G)** The bar graphs demonstrate the average of SIRT1, P-LKB1, P-AMPK, P-ACC, P-HSL, and FAS levels. *P < 0.05, **P < 0.01, *vs* control group, and the data are expressed as mean ± SD (n = 8).

**Figure 5:**
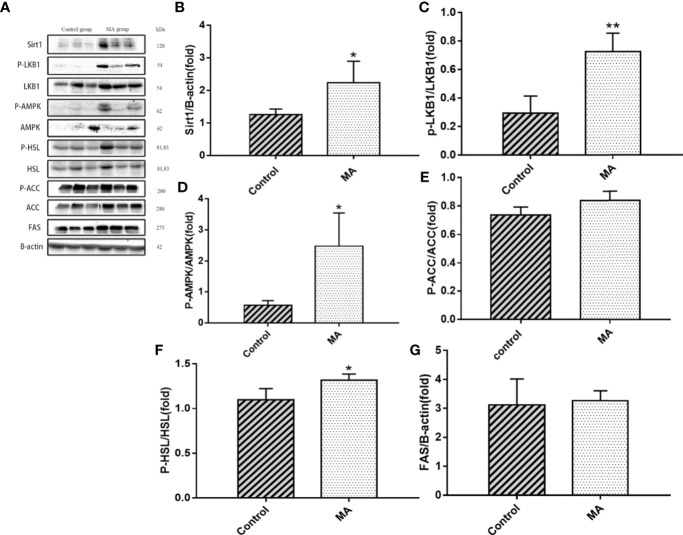
Effects of MA on related factors of epididymal fat. **(A)** The protein expression levels of SIRT1, P-LKB1, LKB1, P-AMPK, AMPK, P-HSL, HSL, P-ACC, ACC, FAS, and β-actin of control group and MA group. **(B–G)** The bar graphs demonstrate the average of SIRT1, P-LKB1, P-AMPK, P-HSL, P-ACC, and FAS levels. *P < 0.05, **P < 0.01, *vs* control group, and the data are expressed as mean ± SD (n = 8).

**Figure 6:**
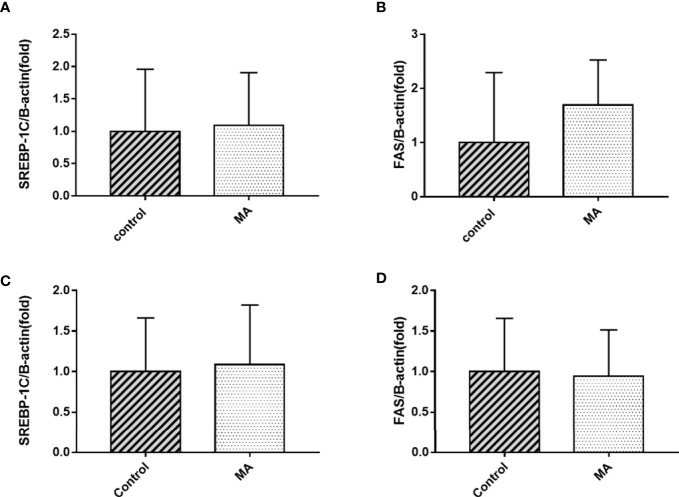
Effects of MA on SREBP1c and FAS mRNA expression in mesenteric fat and epididymal fat. **(A, B)** The expression of SREBP1c and FAS mRNA in mesenteric fat. **(C, D)** The expression of SREBP1c and FAS mRNA in epididymal fat. Data are expressed as mean ± SD (n = 8).

The results indicate that MA treatment can inhibit body weight gain and decrease TG levels of KKay/TaJcl mice, but no significant difference was observed in the visceral fat weight gain between the two groups. To identify the exact mechanism of MA on fat metabolism of KKay/TaJcl mice, the expression of P-AMPK, P-ACC, and P-HSL of abdominal fat, mesenteric fat, and epididymal fat was examined by western blotting (**Figures 3**–**5**). The results showed that MA has no effect on abdominal fat, but it can affect lipogenesis of mesenteric fat through AMPK-ACC signaling pathway, and promote lipolysis of epididymal fat through AMPK-HSL signaling pathway. Subsequently, the upstream proteins of AMPK were detected and revealed that MA significantly promoted the expression of SIRT1 on epididymal fat and mesenteric fat (**Figures 4B**, **5B**), whereas no meaningful effect was observed on FAS and SREBP1c expression (**Figures 4G**, **5G**, **6**). These experimental results and analysis showed that the mechanism of MA might be mediated mainly through SIRT1-AMPK signaling pathway by affecting the mesenteric fat and epididymal fat. However, the mechanism of action on these two tissues is different. MA is mainly mediated through SIRT1-AMPK-ACC signaling pathway to inhibit mesenteric fat lipogenesis, whereas epididymal fat is mainly affected through SIRT1-AMPK-HSL signaling pathway.

### MA Accelerates Fatty Acid Oxidation of Epididymal Fat

**Figure 7:**
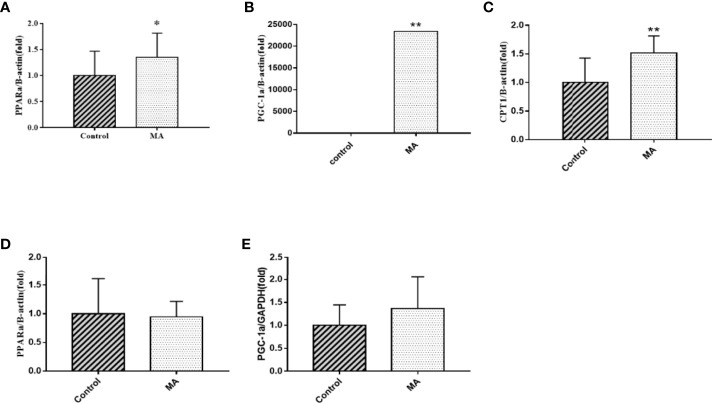
Effects of MA on fatty acid oxidation in epididymal fat and mesenteric fat. **(A)** The expression mRNA levels of PPARα in epididymal fat. **(B)** The expression mRNA levels of PGC-1α in epididymal fat. **(C)** The expression mRNA levels of CPT-1a in epididymal fat. **(D)** The expression mRNA levels of PPARα in mesenteric fat. **(E)** The expression mRNA levels of PGC-1α in mesenteric fat. *P < 0.05, **P < 0.01, *vs* control group, and the data are expressed as mean ± SD (n = 8).

The results showed that MA treatment promoted lipolysis of epididymal fat, and lipolysis was often accompanied with fatty acid oxidation. Moreover, *Brendan N Reid* et al. have pointed out that overexpression of hormone-sensitive lipase (HSL) promoted fatty acid oxidation and stimulated the release of free fatty acids directly (19), and so the factors of epididymal fat that play a key role in fatty acid metabolism was examined by PCR (**Figures 7A–C**). The results revealed that MA treatment significantly improved the expression of CPT-1a, PPARα, and PGC-1α in epididymal fat (P < 0.05). Furthermore, we also detected the expression of PPARα and PGC-1α in the mesenteric fat, but it did not seem to be affected in mesenteric fat after treating with MA (**Figures 7D, E**), suggesting that MA might not accelerate fatty acid oxidation in mesenteric fat.

### MA Inhibits the Expression of AMPK in the Cortex

**Figure 8:**
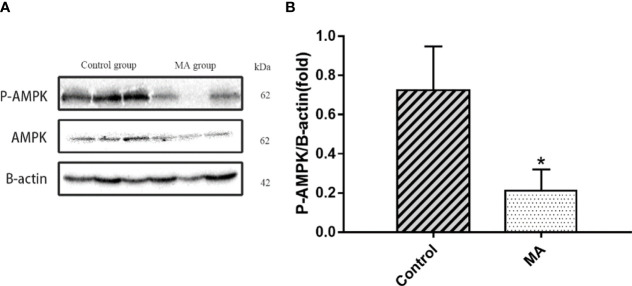
Effects of MA on AMPK in cortex. **(A)** The protein expression levels of P-AMPK, AMPK, β-actin of control group and MA group. **(B)** The bar graphs demonstrate the average of P-AMPK levels in the cortex. *P < 0.05, *vs* control group, and the data are expressed as mean ± SD (n = 8).

Central nervous system is closely related to obesity, and the relationship between hypothalamus and obesity has received widespread attention. However, there is less research on the relationship between cortex and obesity. A pre-clinical study has pointed out that the expression of AMPK in the cortex of obese mice showed hyperphosphorylation, in which the mice were fed with high-fat diet (20). Next, the P-AMPK levels in the MA group were reduced (P < 0.05) when studying the expression of related proteins in the mice cortex (**Figures 8A, B**).

### MA Stimulates the Expression of Thermoregulatory Genes in Brown Fat and Mesenteric Fat

**Figure 9:**
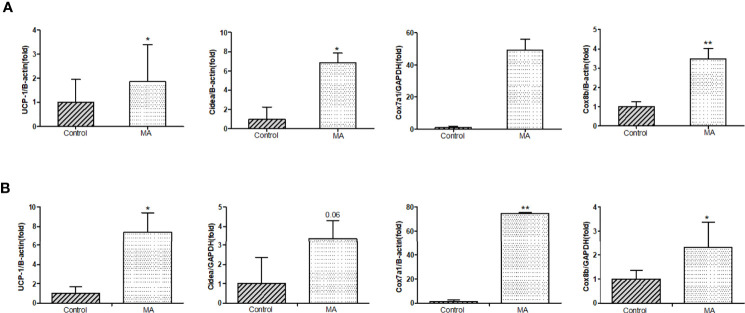
Effects of MA treatment on thermoregulatory genes in mesenteric fat and brown fat. **(A)** The bar graphs demonstrate the average of UCP-1 mRNA, Cidea mRNA, Cox7a1 mRNA, and Cox8b mRNA levels in mesenteric fat between control group and MA group. **(B)** The bar graphs demonstrate the average of UCP-1 mRNA, Cidea mRNA, Cox7a1 mRNA, and Cox8b mRNA levels in brown fat. *P < 0.05, **P < 0.01, *vs* control group, and the data are expressed as mean ± SD (n = 8).

Next, the expression of UCP-1 in brown fat, mesenteric fat, and epididymal fat was investigated. The results have pointed out that MA can significantly increase UCP-1 expression in brown fat and mesenteric fat. Furthermore, we investigated if thermoregulatory genes, such as Cox7a1, Cox8b, and Cidea, were affected, and the results revealed that MA treatment can stimulate the expression of thermoregulatory genes in these two adipose tissues (**Figures 9A, B**).

## Discussion

**Figure 10:**
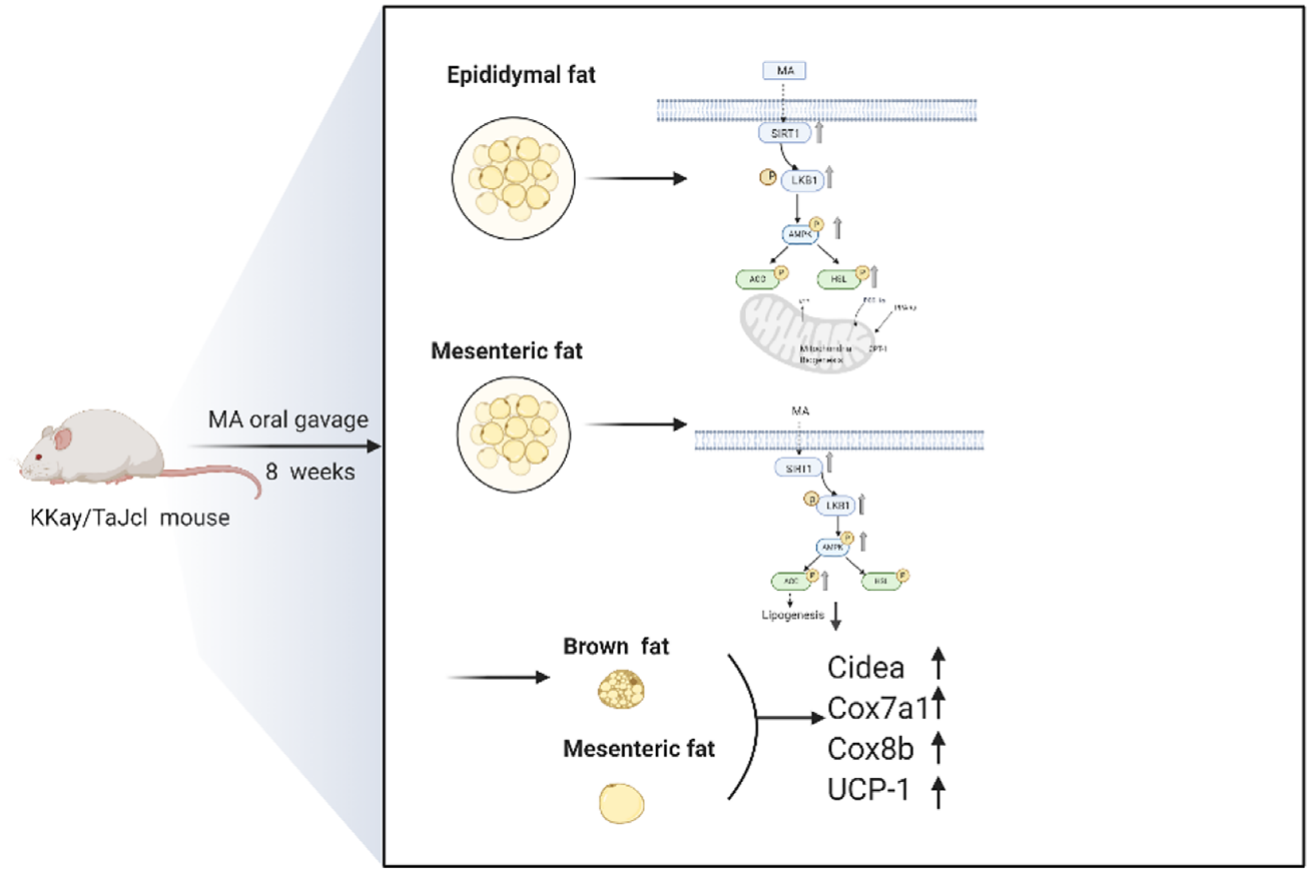
Schematic diagram of the action mechanism of MA.

KKay mice are spontaneous diabetic and obese models that are good for studying the metabolism-related diseases (21, 22). As early as 1988, some scholars have found that the effect of thermogenesis in brown fat is involved in obese KKay mice (23). So we choose this animal model to develop our research. Obesity usually occurs due to excess nutrition and sedentary lifestyle (24), and fat tissue is essential for systemic metabolic homeostasis, and adipocytes can participate in regulating the body metabolism by increasing or decreasing the number or changing the size (25). Obesity can be effectively suppressed by inhibiting the differentiation of adipocytes or promoting the decomposition of adipocytes. Moreover, it is also essential to activate brown adipose tissue and browning of white adipose tissue for anti-obesity treatment (26–28). Our experiment revealed that MA treatment can inhibit weight gain of KKay mice, and it may be at least partially mediated by the following three mechanisms:

First of all, MA can inhibit mesenteric fat lipogenesis. The above results indicated that MA can significantly promote the expression of SIRT1, P-AMPK, and P-ACC in mesenteric fat. SIRT1 is an important regulator of adipose tissue maturation and remodeling. Apart from regulating the gene transcription of white adipose tissue (WAT) to adapt to the systemic energy regulation, it can also affect the differentiation and remodeling of brown adipose tissue (BAT) by regulating the activity of PGC-1α and PPAR-γ factors (29–31). AMPK acts as an energy regulator, and requires activation to exert anti-obesity effects in the surrounding tissues (32, 33). ACC is the downstream factor of AMPK, and it often plays an essential role on fatty acid metabolism (34). Our experimental results showed that MA treatment can promote the activation of SIRT1, AMPK, and ACC in the mesenteric fat tissue, suggesting that mechanism of MA is mainly done through SIRT1-AMPK-ACC signaling pathway for inhibiting lipogenesis in the mesenteric fat tissue (**Figure 10**).

Next, our studies have demonstrated that MA can promote lipolysis and fatty acid oxidation in epididymal fat. As mentioned above, MA can activate SIRT1-AMPK-HSL signaling pathway in epididymal fat. HSL is the downstream factor of AMPK, which in turn affects lipid metabolism by promoting lipolysis (35). Fatty acid oxidation can effectively prevent lipid aggregation and cardiomyopathy in obese mice (36, 37). And PGC-1α acts as a key regulator of mitochondrial biogenesis and function, PPARα is involved in regulating fatty acid and cholesterol metabolism, and CPT 1A is an enzyme that is anchored to the outer mitochondrial membrane (OMM), and it regulates the passage of fatty acids into the mitochondria and interferes with β-oxidation of long-chain fatty acids. These three factors play an important role in the occurrence of fatty acid oxidation (38–40). So, these three factors were tested and shown in **Figure 7**. MA can enhance the expression of these three factors in the epididymal fat (PGC-1α, PPARα, and CPT-1a). All these suggest that MA might affect through SIRT1-AMPK-HSL signaling pathway and fatty acid oxidation in the epididymal fat in order to modify lipid metabolism (**Figure 10**).

In our research, the effect of MA in inhibiting the expression of AMPK in the cortex has been established. Moreover, it can up-regulate UCP-1 and promote mitochondrial function genes expression (Cidea, Cox7a1, and Cox8b) in brown fat as well as mesenteric fat. Mitochondria is rich in BAT, and the physiological function of it is recognized by non-trembling heat, moreover, mitochondrial biogenesis plays a crucial role in generating heat in brown fat (41). This in turn is regarded beneficial to improve obesity by activating brown fat to increase energy expenditure and white fat browning (42). Researchers have found that hypothalamus can stimulate eating and influence the expression of UCP-1 mRNA (43, 44). With increasing research, AMPK played a role in hypothalamus in regulating the appetite and energy expenditure (45, 46). In addition, the inhibition of AMPK in the hypothalamus also regulates BAT heating and WAT browning (47). Some literatures have shown that AMPK level in the cortex is closely related to the occurrence of cognitive dysfunction, and suppression of AMPK activity by pharmacology or heritable gene in the cortex can prevent exogenous amyloid beta exposure or disruption of synaptic plasticity caused by APP/PS1 transgenic mice (48). *Bhumsoo Kim et al*. have proposed that AMPK is increased in the cortex, which might be a risk factor for obesity (20). But the mechanism of action that showed correlation between AMPK in the cortex and obesity has not been investigated till date. Further review of literatures revealed that inhibition of AMPK in the ventral nucleus of the hypothalamus possibly ameliorated obesity by increasing the brown fat product and energy metabolism (49). We tried to check the expression of AMPK in the cortex and UCP-1 in brown fat, mesenteric fat, and epididymal fat. The results have revealed that MA treatment can significantly inhibit the expression of AMPK in the cortex and increase UCP-1 expression, moreover, it also promoted the expression of thermogenic genes in (Cidea, Cox7a1, and Cox8b) brown fat as well as mesenteric fat.

In summary, this is the first study to investigate the effects of MA on the inhibition of body weight gain. Our experimental results showed that MA can effectively promote the expression of SIRT1 and AMPK in mesenteric fat and epididymal fat. Moreover, MA treatment inhibited lipogenesis in mesenteric fat tissue mainly through SIRT1-AMPK-ACC signaling pathway, while promoted lipolysis through SIRT1-AMPK-HSL signaling pathway in epididymal fat. By enhancing the expression of fatty acid oxidation related factors in epididymal fat (PGC-1α, PPARα, and CPT-1a), activation of BAT and WAT browning, suggesting that MA treatment can improve obesity by regulating lipid metabolism and promoting energy expenditure. As is shown above, oral bioavailability of MA is poor (11), but according to one of our previous systematic review, MA may be absorbed and utilized through enterohepatic circulation (50). The current experiment revealed that MA has significant effect on mesenteric fat and epididymal fat, but no detectable effect on abdominal fat was observed. We hypothesize these different therapeutic impacts may be related to the location of different tissues. Mesenteric and epididymal adipose are closer to enterohepatic circulation, the MA concentration might be higher in these parts than that of abdominal adipose. However, there are a few shortcomings in this experiment, which are as follows: we could not detect the core temperature and energy expenditure of the mice in the two groups. The blood lipids and blood glucose indicators in the 4th week were measured using blood from tail vein, whereas in the 8th week, the parameters were measured using aortic blood, and this has caused the abnormal decrease of TG and glucose levels in the 8th week compared with the 4th week. More importantly, though *Centella Asiatica* was reported to have a positive effect on diabetic animals (51), MA showed no effect on glucose levels in our experiment, for which the underlying mechanisms are still not clear and further research are needed here.

## Conclusion

MA treatment can inhibit weight gain by affecting lipid metabolism of visceral fat through different signaling pathways, and it might affect energy metabolism through AMPK levels in the cortex. All these require further verification in the future studies.

## Data Availability Statement

The original contributions presented in the study are included in the article/supplementary material. Further inquiries can be directed to the corresponding authors.

## Ethics Statement

The animal study was reviewed and approved by the Experimental Animal Ethics Committee of the Mukogawa Women’s University in Japan.

## Author Contributions

BS carried out the studies, acquired the data, performed the data analysis, and drafted and revised the manuscript. MH, MK, LW, and LQ participated in the animal experiments. TL and MG were involved in the design and organization of the study, interpreted the results, and revised the manuscript. All authors contributed to the article and approved the submitted version.

## Funding

This work was supported by Mukogawa Women’s University, Beijing International Science and Technology Cooperation Base, and Traditional Chinese Medicine of Beijing Key Laboratory (No. BZ0259) for financial support. The funders have no role in designing study and manuscript writing.

## Conflict of Interest

The authors declare that the research was conducted in the absence of any commercial or financial relationships that could be construed as a potential conflict of interest.

## Abbreviations

PPARα, peroxisome proliferator activated receptor alpha; UCP1, ncoupling protein 1; PGC-1α, peroxisome proliferative activated receptor, gamma, coactivator 1 alpha; Cidea, cell death-inducing DNA fragmentation factor, alpha subunit-like effector A; Cox7a1/8b, cytochrome c oxidase subunit 7a1/8b; CPT1, carnitine palmitoyltransferase 1; SREBP1c, sterol regulatory element-binding transcription factor 1; Sirt1, Sirtuin 1; LKB1, liver kinase B1; AMPK, AMP-activated protein kinase; ACC, acetyl-coenzyme A carboxylase; HSL, hormone-sensitive lipase; FAS, fatty acid synthase; AST, aspartate aminotransferase; ALT, alanine aminotransferase; CHO, cholesterol; TG, triglycerides.
